# *In Vitro* Antiprotozoal Activity of Abietane Diterpenoids Isolated from *Plectranthus barbatus* Andr.

**DOI:** 10.3390/ijms15058360

**Published:** 2014-05-12

**Authors:** Ramzi A. Mothana, Mansour S. Al-Said, Nawal M. Al-Musayeib, Ali A. El Gamal, Shaza M. Al-Massarani, Adnan J. Al-Rehaily, Majed Abdulkader, Louis Maes

**Affiliations:** 1 Department of Pharmacognosy, College of Pharmacy, King Saud University, P.O. Box 2457, Riyadh 11451, Saudi Arabia; E-Mails: msalsaid@ksu.edu.sa (M.S.A.-S.); nalmusayeib@ksu.edu.sa (N.M.A.-M.); aelgamal00@yahoo.com (A.A.E.G.); salmassarani@ksu.edu.sa (S.M.A.-M.); ajalreha@ksu.edu.sa (A.J.A.-R.); 2 Department of Pharmacognosy, College of Pharmacy, Salman Abdulaziz University, Alkharj 11942, Saudi Arabia; E-Mail: mpharm101@hotmail.com; 3 Laboratory for Microbiology, Parasitology and Hygiene (LMPH), Faculty of Pharmaceutical, Biomedical and Veterinary Sciences, Antwerp University, Universiteitsplein 1, B-2610 Antwerp, Belgium; E-Mail: louis.maes@uantwerpen.be

**Keywords:** *Plectranthus barbatus*, antiprotozoal, abietane-type diterpenoids, *Plasmodium*, *Leishmania*, *Trypanosoma*, cytotoxicity

## Abstract

Chromatographic separation of the *n*-hexane extract of the aerial part of *Plectranthus barbatus* led to the isolation of five abietane-type diterpenes: dehydroabietane (**1**); 5,6-didehydro-7-hydroxy-taxodone (**2**); taxodione (**3**); 20-deoxocarnosol (**4**) and 6α,11,12,-trihydroxy-7β,20-epoxy-8,11,13-abietatriene (**5**). The structures were determined using spectroscopic methods including one- and two-dimensional NMR methods. Compounds (**1**)–(**3**) and (**5**) are isolated here for the first time from the genus *Plectranthus*. The isolated abietane-type diterpenes tested *in vitro* for their antiprotozoal activity against erythrocytic schizonts of *Plasmodium falciparum*, intracellular amastigotes of *Leishmania infantum* and *Trypanosoma cruzi* and free trypomastigotes of *T. brucei*. Cytotoxicity was determined against fibroblast cell line MRC-5. Compound (**2**) 5,6-didehydro-7-hydroxy-taxodone showed remarkable activity with acceptable selectivity against *P. falciparum* (IC_50_ 9.2 μM, SI 10.4) and *T. brucei* (IC_50_ 1.9 μM, SI 50.5). Compounds (**3**)–(**5**) exhibited non-specific antiprotozoal activity due to high cytotoxicity. Compound (**1**) dehydroabietane showed no antiprotozoal potential.

## 1. Introduction

The genus *Plectranthus* (Labiatae) represents a large and widespread group of species with a diversity of traditional medicinal uses. The genus comprises a group of around 300 species, distributed in tropical and subtropical areas of Africa, Asia and Australia [1]. One of the most interesting species of this genus is *Plectranthus barbatus* Andr., which is well-known for the treatment of various ailments. A diversity of traditional medicinal uses of *P. barbatus* in India (Hindu and Ayurvedic medicine), East and Central Africa, China, and Brazil have been reported [2]. The majority of uses are for intestinal disturbances and liver fatigue, respiratory disorders, heart diseases and certain central nervous system disorders [2–5]. In previous work [6–8], we screened around 70 medicinal plants from the Arabian Peninsular region (Yemen and Saudi Arabia) for their antiplasmodial, antileishmanial and antitrypanosomal properties. *Plectranthus barbatus* represented one of the more interesting plants for its *in vitro* antiprotozoal effects [8]. The present study on *P. barbatus* specifically deals with bio-guided fractionation, isolation and structural elucidation of abietane diterpenoid constituents.

## 2. Results and Discussion

**Figure 1.**
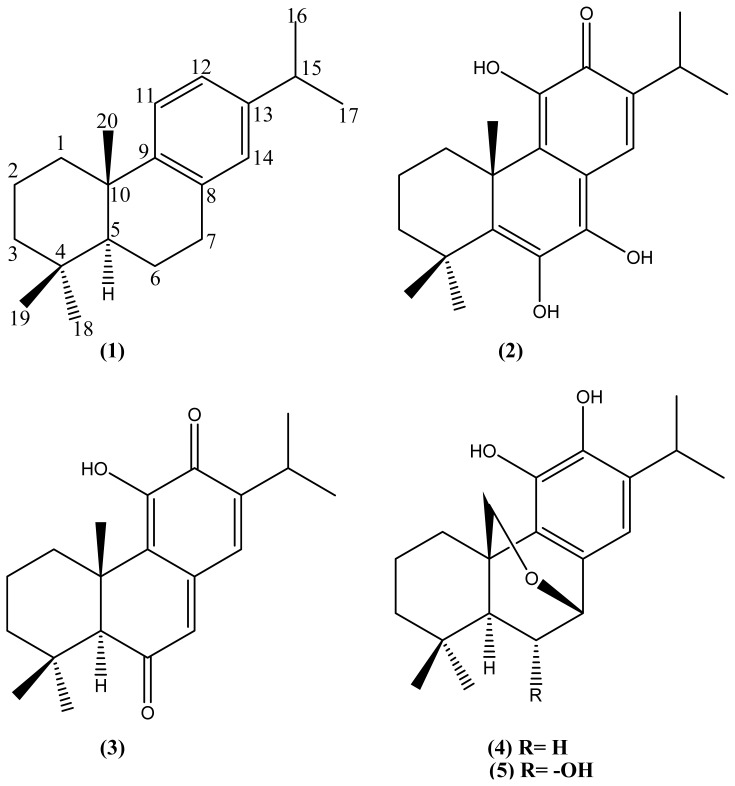
Chemical structures of the isolated abietane diterpenes from *P. barbatus*.

During our previous research for compounds with antiprotozoal activities from medicinal plants [6–8], we found that the extract from *P. barbatus* revealed antiplasmodial, antileishmanial and antitrypanosomal potential [8]. The analysis of the *n*- hexane extract of the aerial part led to the isolation of five abietane-type diterpenes, identified as dehydroabietane (**1**), 5,6-didehydro-7-hydroxy-taxodone (**2**), taxodione (**3**), 20-deoxocarnosol (**4**) and 6α,11,12,-trihydroxy-7b,20-epoxy-8,11,13-abietatriene (**5**). The isolated diterpenoid compounds (Figure 1) were previously isolated from different plant species including *Plectranthus*, *Salvia* and *Taxodium* species and identified by comparison of their spectra data with those reported in the literature [9–18].

### 2.1. Spectral Data

Compound (**4**) was revealed to have the molecular formula C_20_H_28_O_3_, by HR-ESIMS (*m*/*z* 316.9894) with 7 degree of unsaturation. The UV absorption at 320 and 282 nm indicated the presence of the benzene ring. In the IR spectrum, a hydroxyl group absorption was observed at 3400 cm^−1^ and absorptions at 1600 and 1510 cm^−1^ for the aromatic ring. Twenty carbon signals were observed in the ^13^C-NMR and DEPT-experiment, six of them appeared in the aromatic area and indicated the presence of three double bonds at δ 143.5, 142.0, 134.7, 133.9, 129.6 and 112.9 assigned for carbons 12, 11, 8, 13, 9 and 14 respectively (Table 1). A singlet aromatic proton at δ_H_ 6.60 in ^1^H-NMR spectrum postulates the presence of a penta-substituted aromatic ring. In addition, it showed the presence of one hydroxymethylene group at δ_H_ 4.31 and 3.01 (2H, d, *J* = 8.5 Hz), downfield oxymethine at δ_H_ 4.67 (br t, *J* = 3.9 Hz) assigned for H7. The presence of an isopropyl group linked to a quaternary carbon was supported by the signals at δ_H_ 3.25 (1H, m, H15), 1.20 (3H, br d, *J* = 6.0 Hz, CH_3_-16) and 1.21 (3H, br d, *J* = 6.0 Hz, CH_3_-17). Besides the isopropyl group, the presence of two tertiary methyl groups at δ_H_ 0.87 (3H, s) and 1.15 (3H, s) in the ^1^H-NMR and 20 carbon signal in the ^13^C-NMR spectra (Table 1) revealed an abietane diterpenoid structure [9,10]. The absence of a tertiary methyl group (C20) at C10 frequently present in abietane-type diterpene and the presence of germinal hydroxymethelene protons at δ_H_ 4.31 and 3.01 (2H, d, *J* = 8.5 Hz) suggests that methyl group 20 was replaced by hydroxymethylene. The measurement of long range HMBC experiment was useful in determining the final structure where two and three bond correlations were observed from the methylene protons (H_2_O) (δ_H_ 4.31, 3.01) to C5, C7, C9 and C10, from H14 at δ_H_ 6.60 and the carbons at δ_C_ 72.7 (C7) and δ_C_ 129.6 (C9); and between the proton resonance at δ_H_ 4.67 (H7) and the carbons at δ_C_ 44.6 (C5) and δ_C_ 129.6 (C9). The above findings confirmed that Compound (**4**) is an abietane-type diterpene with an ether linkage between C20 and C7. The presence of such a type of ether linkage is uncommon in the plants belonging to family Lamiaceae. Comparing the above mentioned NMR data, MS and other spectral finding with those reported for 20-deoxocarnosol proved that both compounds were identical [9,10].

Compound (**5**) was obtained as colorless crystals. The HR-ESIMS gave a molecular ion peak at *m*/*z* 332.9893, corresponding to a molecular formula of C_20_H_28_O_4_ with 16 mass units more than Compound (**4**). The IR spectrum showed absorption bands, like Compound (**4**), at 3400 (OH), and 1620 and 1500 cm^−1^ (aromatic). The presence of an aromatic ring was supported by the UV data (λ_max_ 210 and 282, 320 nm). The ^13^C-NMR and DEPT-experiment were in part similar to those of Compound (**4**), the main difference was the decrease number of methylene protons by one (3 in Compound (**5**) and 4 in Compound (**4**)) and the subsequent increase in the number of methine protons by one (5 in Compound (**5**) and 4 in Compound (**4**)). It showed, like Compound (**4**), 20 carbon signals, six of them appeared at δ_C_ 144.2 (C12), 141.7 (C11), 134.8 (C13), 129.9 (C8), 129.6 (C9) and 116.6 (C14) ascribed for pentasubstutited aromatic ring (Table 1). The major difference was the presence of an extra downfield signal at δ_H_ 4.09 (br s, δ_C_ 69.9) indicating a substitution with OH-group. The rest of ^1^H-NMR spectral data was almost identical to Compound (**4**) and confirmed the presence of abietane-type diterpene with an aromatic ring and ether linkage between C20 and C7. The extra hydroxyl group was positioned at C6 through the observed long range cross peaks correlations in HMBC experiment between proton at δ 4.09 (br s, H6) and C4, C5 and C10; between hydroxymethylene group at δ_H_ 4.11 and C5, C7, C9 and C10; between the multiple proton at δ 3.25 (H15) and C12, C13 and C14; between the proton at δ H 6.69 (H14) and C9 and C12; between the proton at δ 4.47 (H7, ether linkage proton) and C5, C6 and C9. The rest of HMBC correlations were closely similar to those of Compound (**4**). The orientation for hydroxyl group at C6 was confirmed to be α by comparing ^13^C-NMR chemical shift for C5, C6 and C7 with those reported for 6α,11,12,-trihydroxy-7β,20-epoxy-8,11,13-abietatriene, recently isolated from *Premna obtusifolia* (Verbenaceae) [11]. It is worth noting that similar structural compounds have been isolated from *Coleus eskirolii* and *Salvia aspera* [12,13] and identified as esquirolin D and 6-epi-demethylesquirolin D. However in esquirolin D, the hydroxyl group at position 6 was β-orientated rather than α as in Compound (**5**) and in 6-epi-demethylesquirolin D.

The other three known diterpenes (**1**) (dehydroabietane) [14], (**2**) (5,6-didehydro-7-hydroxy-taxodone) [15] and (**3**) (taxodione) [16–18] were previously isolated from other plant species particularly from *Salvia* and *Taxodium* species. The chemical structures were determined by comparison of NMR spectral data with published data.

### 2.2. Antiprotozoal Activity

*Plectranthus barbatus* was previously shown to have antiplasmodial and antitrypanosomal activity [8], which encouraged us to fractionate and isolate some of its constituents with antiprotozoal activity against *P. falciparum*, *L. infantum*, *T. cruzi* and *T. brucei* as well as with cytotoxic activity against MRC-5 cells (Table 2). To the best of our knowledge, this study represents the first report on antiplasmodial, antileishmanial and antitrypanosomal activities of the isolated diterpenoids (**1**), (**2**), (**4**) and (**5**) (Table 2).

5.6-Didehydro-7-hydroxy-taxodone (**2**) showed interesting activity and selectivity against *P. falciparum* (IC_50_ 9.2 μM, SI 10.4) and *T. brucei* (IC_50_ 1.9 μM, SI 50.5). Taxodione (**3**), 20-deoxocarnosol (**4**) and 6α,11,12,-trihydroxy-7β,20-epoxy-8,11,13-abietatriene (**5**) showed non-specific activity against all protozoa species with IC_50_-values between 6.0 and 31.6 μM but with high cytotoxicity against MRC-5 cells (IC_50_-values between 5.7 and 22.6 μM). Dehydroabietane (**1**) was inactive against all species (IC_50_ > 100 μM).

These results are in agreement with literature data on diterpenoids isolated from other plant species [19–25]. Our result for taxodione (**3**) was consistent with that obtained by Machumi *et al.* [19] who reported antileishmanial and antimalarial activities for taxodione isolated from the roots of *Clerodendrum eriophyllum*. Van Zyl *et al.* [20] reported on seven abietane diterpenes from the *Plectranthus* species *P. hadiensis*, *P. lucidus*, *P. ecklonii*, *P. purpuratus* subsp. *purpuratus* and *P. purpuratus* subsp. *Tongaensis*. All seven compounds were tested for their antiplasmodial activity and for their ability to inhibit β-haematin formation. Overall, they exhibited activity with IC_50_ values ranging from 3.11 to 14.65 μM in inhibiting β-haematin formation; however, the cytotoxicity profile indicated a low degree of specificity. Sairafianpour *et al.* [21] reported the isolation of diterpenoid 1,2-quinones (tanshinones) from the roots of *Perovskia abrotanoides* which exhibited *in vitro* antileishmania activity with IC_50_ values in the range of 18–47 μM. These isolated tanshinones inhibited also the growth of cultured 3D7 strain of *P. falciparum*, KB-3-1-human carcinoma cell line, KBV1 cell line and human lymphocytes with IC_50_ values in the range of 5–45 μM [21]. A bioassay-guided fractionation of the extract of the roots of *Salvia cilicica* led to the isolation of antileishmanial diterpenoids [22] in which the isolated 7-hydroxy-12-methoxy-20-nor-abieta-1,5(10),7,9,12-pentaen-6,14-dione and abieta-8,12-dien-11,14-dione (12-deoxy-royleanone), together with oleanolic acid, ursolic acid, ferruginol, inuroyoleanol and cryptanol were found to be potent against amastigote form of *L. donovani* and *L. major* with IC_50_ values of 120–290 nM. Similar antiparasitic and nematicidal activity was found for *Juniperus procera* by Samoylenko *et al.* [23] where bioguided fractionation of *J. procera* berries led to the isolation of abietane, pimarane and labdane diterpenes which inhibited *L. donovani* promastigotes with IC_50_ values of 3.5–4.6 μg/mL [23]. Similar results were reported on natural or synthesized abietane diterpenoids with trypanocidal and leishmanicidal activities isolated from *Craniolaria annua* and other plant species [24,25].

The lipophilic nature of the abietane diterpenes enables easy transport across the parasitic membranes to accumulate in the parasitic food vacuole [20]. Comparing the IC_50_ values of the isolated abietane diterpenes **1**–**5**, some structure-activity relationships may be suggested. The results suggest that the antiprotozoal activity depends on oxygenated and dehydrogenated chromophoric systems through rings B and C since dehydroabietane (**1**), without oxygen functions in ring B and C as well as dehydrogenations in ring B, had no activity against all protozoal strains. The structural analysis of the diterpenes **2**–**5** allow us to conclude that the quinone-structure at C6, C7, C11 and C12 apparently increases the antiprotozoal activity as well as the antifungal and antibacterial activities of several abietane-type diterpene quinones [17]. Quinones should not be the only chemical group required for antiprotozoal activity. A comparison of Compounds (**4**) and (**5**) as well as Compounds (**2**) and (**3**) revealed that hydroxylation at C6, C7, C11 and C12 was translated into strong antiprotozoal and cytotoxic activity. Moreover, a comparison of Compounds (**2**) and (**3**) showed an en-ol-structure at C6 in Compound (**2**) instead of oxo-structure in Compound (**3**) which is apparently translated into more selectivity for 5,6-didehydro-7-hydroxy-taxodone (**2**). As most diterpenes are known to combine antiprotozoal activity with high cytotoxicity [26], 5.6-didehydro-7-hydroxy-taxodone (**2**) may be withheld as the better antiprotozoal agent in view of the more favorable selectivity indices.

## 3. Experimental Section

### 3.1. General Experimental Procedures

The UV and IR spectra were recorded on UV-1601-PC (Shimadzo, Koyoto, Japan) and JASCO 320-A spectrometers (JASCO Germany GmbH, Gross-Umstadt, Germany). The ^1^H-, ^13^C-NMR and 2D-NMR spectra were recorded on a Bruker AMX-500 spectrometer (Bruker, Faellanden, Switzerland) with tetramethylsilane (TMS) as internal standard. Chemical shifts are given in ppm (δ) relative to tetramethylsilane internal standard and scalar coupling constants (*J*) are reported in Hertz. Jeol JMS-700 High Resolution Mass Spectrophotometer (JEOL (Germany) GmbH, Muenchen, Germany) was used for the mass determination. Electron Impact mode of ionization was used, keeping ionization energy of 70 eV. Resolution was set up to 10 k. A direct probe was used with temperature ramp setting, initial temperature 50 °C rise with rate of 32 °C per minute and final temperature set up to 350 °C. Thin layer chromatography (TLC) was performed on precoated silica gel F_254_ plates (E. Merck, Darmstadt, Germany); detection was done at 254 nm and by spraying with *p*-anisaldehyde/H_2_SO_4_ reagent. All chemicals were purchased from Sigma Chemical Company (St. Louis, MO, USA).

### 3.2. Plant Materials

Aerial part (leaves, stems and flowers) of *P. barbatus* was collected from Wadi Gama in Taif province of Saudi Arabia in March 2010 and identified at the Pharmacognosy Department, College of Pharmacy, King Saud University. A voucher specimen (Voucher # P-15451) was deposited at the Pharmacognosy Department, College of Pharmacy, King Saud University.

### 3.3. Extraction and Isolation

The air-dried and powdered aerial part of *P. barbatus* (1 kg) was extracted by maceration with 70% ethanol (5 × 2 L) at room temperature. The combined obtained ethanolic extract was filtered and concentrated under reduced pressure at 40 °C using a rotary evaporator. The dried ethanolic extract (65 g) was subsequently redissolved in 30% ethanol (200 mL) and partitioned successively for several times with *n*-hexane (3 × 200 mL), chloroform (3 × 200 mL) and *n*-butanol (3 × 200 mL) to provide the corresponding extracts. Each extract was tested for its antiprotozoal activity. Consequently, it was shown that both activities resided predominantly in the hexane and chloroform extracts. Hence, the *n*-hexane extract (26 g) was subjected to column chromatography on a pre-packed silica gel column (35 mm i.d. × 350 mm) to give 15 fractions. The elution was performed with a gradient of hexane:acetone (10:1) to pure acetone. TLC analysis of the fractions with anisaldehyde/sulfuric acid and heating at 100 °C allowed the constitution of 15 fractions. Fraction 3 (4.5 g) was separated on a RP18 column with methanol:acetonitile (1:9) to afford 485 mg of colorless oil (Compound (**1**)). Fraction 14 (350 mg) was subjected to a silica gel column chromatography using dichloromethane:methanol (100:1) as a solvent to afford two subfractions (Fraction 14a and 14b) Fraction 14a afforded Compound (**2**) which required a further separation on the chromatotron (centrifugal TLC) (silica gel 60, 0.04–0.06 mm, 1 mm and methanol:DCM, 0.5:99.5) to give 36 mg of yellow crystalline powder (Compound (**2**)). The purification of fraction 14b on a silica gel column with acetone:hexane (1:20) as eluent gave an orange viscous Compound (**3**) (24 mg). Fraction 15 (2.8 g) was separated on a silica gel column with acetone:hexane (1:20) as eluent to afford a subfraction containing Compound (**4**) and Compound (**5**). Both compounds required a further purification on a chromatotron (Silica gel, 0.04–0.06 mm, 1 mm, EtoAc:dichloromethane, (2:8) to give two colorless crystalline powders, namely Compound (**4**) (395 mg) and Compound (**5**) (42 mg).

### 3.4. Biological Assays

The integrated panel of microbial screens and standard screening methodologies were adopted as previously described [27]. All assays were performed in triplicate, at the Laboratory of Microbiology, Parasitology and Hygiene at the University of Antwerp, Belgium. Plant extracts were tested at 5 concentrations (64, 16, 4, 1 and 0.25 μg/mL) to establish a full dose-titration and determination of the IC_50_ (inhibitory concentration 50%). The concentration of DMSO did not exceed 0.5%. The selectivity antiprotozoal potential was assessed by simultaneous evaluation of cytotoxicity on a fibroblast (MRC-5) cell line. The criterion for activity was an IC_50_ <10 μg/mL (<5 μg/mL for *T. brucei*) and a selectivity index of ≥4.

#### 3.4.1. Antileishmanial Activity

*L.infantum* MHOM/MA(BE)/67 amastigotes were collected from the spleen of an infected donor hamster and used to infect primary peritoneal mouse macrophages. To determine *in vitro* antileishmanial activity, 3 × 10^4^ macrophages were seeded in each well of a 96-well plate. After 2 days outgrowth, 5 × 10^5^ amastigotes/well were added and incubated for 2 h at 37 °C. Pre-diluted plant extracts were subsequently added and the plates were further incubated for 5 days at 37 °C and 5% CO_2_. Parasite burdens (mean number of amastigotes/macrophage) were microscopically assessed after Giemsa staining, and expressed as a percentage of the blank controls without plant extract.

#### 3.4.2. Antiplasmodial Activity

Chloroquine-resistant *P. falciparum* 2/K 1-strain was cultured in human erythrocytes O^+^ at 37 °C under a low oxygen atmosphere (3% O_2_, 4% CO_2_, and 93% N_2_) in RPMI-1640, supplemented with 10% human serum. Infected human red blood cells (200 μL, 1% parasitaemia, 2% haematocrit) were added to each well and incubated for 72 h. After incubation, test plates were frozen at −20 °C. Parasite multiplication was measured by the Malstat method [27,28].

#### 3.4.3. Antitrypanosomal Activity

*Trypanosoma brucei* Squib-427 strain (suramin-sensitive) was cultured at 37 °C and 5% CO_2_ in Hirumi-9 medium [29], supplemented with 10% fetal calf serum (FCS). About 1.5 × 10^4^ trypomastigotes/well were added to each well and parasite growth was assessed after 72 h at 37 °C by adding resazurin [30]. For Chagas disease, *T. cruzi* Tulahuen CL2 (benznidazole-sensitive) was maintained on MRC-5 cells in minimal essential medium (MEM) supplemented with 20 mM l-glutamine, 16.5 mM sodium hydrogen carbonate and 5% FCS. In the assay, 4 × 10^3^ MRC-5 cells and 4 × 10^4^ parasites were added to each well and after incubation at 37 °C for 7 days, parasite growth was assessed by adding the β-galactosidase substrate chlorophenol red β-d-galactopyranoside [31]. The color reaction was read at 540 nm after 4 h and absorbance values were expressed as a percentage of the blank controls.

#### 3.4.4. Cytotoxicity Assay

MRC-5 SV2 cells were cultivated in MEM, supplemented with l-glutamine (20 mM), 16.5 mM sodium hydrogen carbonate and 5% FCS. For the assay, 10^4^ MRC-5 cells/well were seeded onto the test plates containing the pre-diluted sample and incubated at 37 °C and 5% CO_2_ for 72 h. Cell viability was assessed fluorimetrically after 4 h of addition of resazurin. Fluorescence was measured (excitation 550 nm, emission 590 nm) and the results were expressed as % reduction in cell viability compared to control.

## 4. Conclusions

In conclusion, five Compounds (**1**)–(**5**) belonging to abietane-type diterpenes were isolated from the aerial part of *Plectranthus barbatus*. Compounds (**1**)–(**3**) and (**5**) were isolated here for the first time from the genus *Plectranthus*. The antiprotozoal activity against *P. falciparum*, *L. infantum*, *T. brucei* and *T. cruzi* is being reported for the first time for four of the isolated diterpenes. Compound (**2**) 5,6-didehydro-7-hydroxy taxodone showed moderate activity against *P. falciparum* and *T. brucei* with acceptable selectivity. Compounds (**3**)–(**5**) exhibited a high antiprotozoal activity but this was due to high cytotoxicity. Compound (**1**) dehydroabietane showed no antiprotozoal potential. These findings stress the importance of structure-activity relationships for biological and toxicological properties of isolated plant constituents whereby potency and selectivity are combined *in vitro* endpoint parameters.

## Figures and Tables

**Table 1.** ^1^H- and ^13^C-NMR Data of Compounds (**1**)–(**5**) (500 MHz for ^1^H- and ^13^C-NMR, (**1**), (**3**) in CDCl_3_ and (**2**), (**4**), (**5**) in CD_3_OD).

Position	Compound (1)		Compound (2)		Compound (3)		Compound (4)		Compound (5)	
				
	δ_H_	δc	δ_H_	δc	δ_H_	δc	δ_H_	δc	δ_H_	δc
1	3.03 m	30.7	3.16 m, 1.59 m	30.8	2.88 m, 1.65 m	37.0	2.73 m, 2.03 m	31.9	2.77 m, 2.15 br d, *J* = 14.5 Hz	31.3
2	1.79 m	19.5	1.84 m	18.7	1.68 m, 1.28 m	18.5	1.60 m	20.1	1.70 m	20.0
3	1.60 m, 1.40 m	41.9	2.03 m, 1.37 m	35.6	3.07 m	42.5	1.54 m, 1.28 m	42.5	1.62 m, 154 m	42.7
4	-	33.6	-	37.5	-	32.8	-	34.9	-	34.6
5	1.50 m	50.5	-	144.4	2.52 br s	62.9	1.45 m	44.6	1.17 br s	56.0
6	2.4 br d, *J* = 12.5 Hz m	39.0	-	144.8	-	201.0	2.01 m, 1.54 m	31.4	4.09 br s	69.9
7	1.99 m, 1.72 m	19.3	-	149.5	6.22 s	134.0	4.67 br t, *J* = 3.9 Hz	72.7	4.47 d, *J* = 3.5 Hz	75.7
8	-	135.0	-	121.6	-	140.0	-	134.7	-	129.9
9	-	147.7	-	144.5	-	125.6	-	129.6	-	129.6
10	-	37.6	-	42.2	-	42.9	-	41.1	-	42.7
11	7.12 d, *J* = 7.8 Hz	123.9	-	140.2	-	145.0	-	142.0	-	141.7
12	7.31 d, *J* = 7.9 Hz	124.4	-	181.8	-	181.7	-	143.5	-	144.2
13	-	145.5	-	135.8	-	145.3	-	133.9	-	134.8
14	7.03 br s	126.9	6.65 s	116.6	6.89 s	136.2	6.60 s	112.9	6.69 s	116.6
15	2.95 m	33.6	3.27 m	27.6	3.00 m	27.1	3.25 m	27.9	3.25 m	28.0
16	1.48 s	24.2	1.10 s	23.1	1.08 d, *J* = 7.0 Hz	21.2	1.20 br d, *J* = 6.0 Hz	23.5	1.34 t, *J* = 6.0 Hz	23.5
17	1.50 s	24.2	1.15 s	23.2	1.10 d, *J* = 7.0 Hz	21.6	1.21 br d, *J* = 6.0 Hz	23.4	1.22 t, *J* = 6.0 Hz	23.4
18	1.19 s	33.5	1.61 s	27.9	1.04 s	33.3	0.87 s	33.6	1.03 s	34.2
19	1.20 s	21.8	1.41 s	28.5	1.20 s	21.8	1.15 s	21.6	1.14 s	23.0
20	1.45 s	25.1	1.42 s	28.0	1.20 s	22.1	4.31 d, *J* = 8.5 Hz, 3.01 d, *J* = 8.5 Hz	70.0	2.91 br d, *J* = 8.2 Hz, 4.11 br d, *J* = 8.2 Hz	68.5

**Table 2.** Antiprotozoal activity and cytotoxicity (IC_50_ in μM) of the isolated compounds from *P. barbatus*.

Compound	*P. falciparum*		*L. infantum*		*T. cruzi*		*T. brucei*		MRC-5
				
	IC_50_	SI	IC_50_	SI	IC_50_	SI	IC_50_	SI	IC_50_
Compound (**1**)	123.7 ± 4.7	1.9	>237.0	> 1	>237.0	>1	>237.0	>1	>237.0
Compound (**2**)	9.2 ± 0.6	10.4	25.7 ± 1.5	3.7	25.7 ± 1.5	3.7	1.9 ± 0.4	50.5	96.2 ± 5.8
Compound (**3**)	8.5 ± 0.7	2.6	25.7 ± 2.3	-	25.7 ± 2.3	-	9.8 ± 0.7	2.3	22.6 ± 1.3
Compound (**4**)	11.1 ± 0.6	-	25.6 ± 1.2	-	25.6 ± 1.2	-	6.0 ± 0.8	1.0	6.0 ± 0.3
Compound (**5**)	31.6 ± 1.9	-	24.4 ± 3.2	-	24.4 ± 3.2	-	15.9 ± 1.4	-	5.7 ± 0.9
Chloroquine	0.04 ± 0.01		-		-		-		-
Miltefosine	-		2.4 ± 0.8		-		-		-
Benznidazole	-		-		2.5 ± 0.6		-		-
Melarsoprol	-		-		-		0.005 ± 0.001		-
Tamoxifen	-		-				-		10.5 ± 2.5

SI: Selectivity index.

## References

[1] Lukhoba C.W., Simmonds M.S.J., Paton A.J. (2006). *Plectranthus*: A review of ethnobotanical uses. J. Ethnopharmacol.

[2] Alasbahi R.H., Melzig M.F. (2010). *Plectranthus barbatus*: A review of Phytochemistry, ethnobotanical uses and pharmacology—Part 1. Planta Med.

[3] Dubey M.P., Srimal R.C., Nityanand S., Dhawan B.N. (1981). Pharmacological studies on coleonol, a hypotensive diterpene from *Coleus forskohlii*. J. Ethnopharmacol.

[4] Zelnik R., Lavie D., Levy E.C., Wang A.H.J., Paul I.C. (1977). Barbatusin and cyclobutatusin, two novel diterpenoids from *Coleus barbatus* Bentham. Tetrahedron.

[5] Dubey M.P., Srimal R.C., Patnaik G.K., Dhawan B.N. (1974). Hypotensive and spasmolytic activities of coleonol, active principle of *Coleus forskohlii* Briq. Indian J. Pharmacol.

[6] Mothana R.A., Al-Musayeib N.M., Matheeussen A., Cos P., Maes L. (2012). Assessment of the *in vitro* antiprotozoal and cytotoxic potential of 20 selected medicinal plants from the island of Soqotra. Molecules.

[7] Al-Musayeib N.M., Mothana R.A., Al-Massarani S., Matheeussen A., Cos P., Maes L. (2012). Study of the *in vitro* antiplasmodial, antileishmanial and antitrypanosomal activities of medicinal plants from Saudi Arabia. Molecules.

[8] Al-Musayeib N.M., Mothana R.A., Matheeussen A., Cos P., Maes L. (2012). *In vitro* antiplasmodial, antileishmanial and antitrypanosomal activities of selected medicinal plants used in the traditional Arabian Peninsular region. BMC Complement Altern. Med.

[9] Topcu G., Ulubelen A. (1996). Abietane and rearranged abietane diterpenes from *Salvia montbretii*. J. Nat. Prod.

[10] Kelecom A. (1984). An abietane diterpene from the Labiate Coleus barbatus. Phytochemistry.

[11] Salae A.-W., Rodjun A., Karalai C., Ponglimanont C., Chantraprommaa S., Kanjana-Opas A., Tewtrakul S., Fun H.-K. (2012). Potential anti-inflammatory diterpenes from *Premna obtusifolia*. Tetrahedron.

[12] Li C., Lin Z., Zheng H., Zhang J., Sun H. (1992). The chemical Constituents of *Coleus esquirolii*. Acta Bot. Yunnanica.

[13] Esquivel B., Flores M., Hernández-Ortega S., Toscano R.A., Ramamoorthy T.P. (1995). Abietane and icetexane diterpenoids from the roots of *Salvia aspera*. Phytochemistry.

[14] Miguel del Corral J.M., Gordaliza M., Salinero M.A., San Feliciano A. (1994). ^13^C-NMR Data for abieta-8,11,13-triene diterpenoids. Magn. Reson. Chem.

[15] Luis J.G., Grillo T.A. (1993). New diterpenes from *Salvia munzii*: Chemical and biogenetic aspects. Tetrahedron.

[16] Hirasawa Y., Izawa E., Matsuno Y., Kawahara N., Goda Y., Morita H. (2007). Taxodistines A and B, abietane-type diterpenes from *Taxodium distichum*. Bioorg. Med. Chem. Lett.

[17] Kusumoto N., Ashitani T., Murayama T., Ogiyama K., Takahashi K. (2010). Antifungal abietane-type diterpenes from the cones of *Taxodium distichum* Rich. J. Chem. Ecol.

[18] Gandomkar S., Yousefi M., Habibi Z., As’habi M.A. (2012). A new triterpene from *Salvia xanthocheila* Boiss. Nat. Prod. Res.

[19] Machumi F., Samoylenko V., Yenesew A., Derese S., Midiwo J.O., Wiggers F.T., Jacob M.R., Tekwani B.L., Khan S.I., Walker L.A. (2010). Antimicrobial and antiparasitic abietane diterpenoids from the roots of *Clerodendrum eriophyllum*. Nat. Prod. Commun.

[20] Van Zyl R.L., Khan F., Edwards T.J., Drewes S.E. (2008). Antiplasmodial activities of some abietane diterpenes from the leaves of five *Plectranthus* species. S. Afr. J. Sci.

[21] Sairafianpour M., Christensen J., Staerk D., Budnik B.A., Kharazmi A., Bagherzadeh K., Jaroszewski J.W. (2001). Leishmanicidal, antiplasmodial, and cytotoxic activity of novel diterpenoid 1,2-quinones from *Perovskia abrotanoides*: New source of tanshinones. J. Nat. Prod.

[22] Tan N., Kaloga M., Radtke O.A., Kiderlen A.F., Oksüz S., Ulubelen A., Kolodziej H. (2002). Abietane diterpenoids and triterpenoic acids from *Salvia cilicica* and their antileishmanial activities. Phytochemistry.

[23] Samoylenko V., Dunbar D.C., Gafur M.A., Khan S.I., Ross S.A., Mossa J.S., El-Feraly F.S., Tekwani B.L., Bosselaers J., Muhammad I. (2008). Antiparasitic, nematicidal and antifouling constituents from *Juniperus* berries. Phytother. Res.

[24] Herrera J.C., Troncone G., Henríquez D., Urdaneta N. (2008). Trypanocidal activity of abietane diterpenoids from the roots of *Craniolaria annua*. Z. Naturforsch. C.

[25] Ramírez-Macías I., Marín C., Es-Samti H., Fernández A., Guardia J.J., Zentar H., Agil A., Chahboun R., Alvarez-Manzaneda E., Sánchez-Moreno M. (2012). Taiwaniaquinoid and abietane quinone derivatives with trypanocidal activity against *T. cruzi* and *Leishmania* spp. Parasitol. Int.

[26] Marques C.G., Pedro M., Simões M.F.A., Nascimento M.S.J., Pinto M.M.M., Rodríquez B. (2002). Effect of abietane diterpenes from *Plectranthus grandidentatus* on the growth of human cancer cell lines. Planta Med.

[27] Cos P., Vlietinck A.J., Berghe D.V., Maes L. (2006). Anti-infective potential of natural products: How to develop a stronger *in vitro* proof-of-concept. J. Ethnopharmacol.

[28] Makler M.T., Ries J.M., Williams J.A., Bancroft J.E., Piper R.C., Hinrichs D.J. (1993). Parasite lactate dehydrogenase as an assay for *Plasmodium falciparum* drug sensitivity. Am. J. Trop. Med. Hyg.

[29] Hirumi H., Hirumi K. (1989). Continuous cultivation of *Trypanosoma brucei* blood stream forms in a medium containing a low concentration of serum protein without feeder cell layers. J. Parasitol.

[30] Raz B., Iten M., Grether-Buhler Y., Kaminsky R., Brun R. (1997). The Alamar Blue asssay to determine drug sensitivity of African trypanosomes (*T. b. rhodesiense, T. b. gambiense*) *in vitro*. Acta Trop.

[31] Buckner F.S., Verlinde C.L., la Flamme A.C., van Voorhis W.C. (1996). Efficient technique for screening drugs for activity against *Trypanosoma cruzi* using parasites expressing beta-galactosidase. Antimicrob. Agents Chemother.

